# Validation of a method for in vivo 3D dose reconstruction in SBRT using a new transmission detector

**DOI:** 10.1002/acm2.12103

**Published:** 2017-06-02

**Authors:** Yuji Nakaguchi, Takeshi Ono, Masato Maruyama, Yoshinobu Shimohigashi, Yudai Kai

**Affiliations:** ^1^ Department of Radiological Technology Kumamoto University Hospital Kumamoto Japan; ^2^ Faculty of Life Sciences Kumamoto University Kumamoto Japan

**Keywords:** in vivo, QA, SBRT, spatial resolution, transmission detector

## Abstract

Stereotactic body radiation therapy (SBRT) involves the delivery of substantially larger doses over fewer fractions than conventional therapy. Therefore, SBRT treatments will strongly benefit patients using vivo patient dose verification, because the impact of the fraction is large. For in vivo measurements, a commercially available quality assurance (QA) system is the COMPASS system (IBA Dosimetry, Germany). For measurements, the system uses a new transmission detector (Dolphin, IBA Dosimetry). In this study, we evaluated the method for in vivo 3D dose reconstruction for SBRT using this new transmission detector. We confirmed the accuracy of COMPASS with Dolphin for SBRT using multi leaf collimator (MLC) test patterns and clinical SBRT cases. We compared the results between the COMPASS, the treatment planning system, the Kodak EDR2 film, and the Monte Carlo (MC) calculations. MLC test patterns were set up to investigate various aspects of dose reconstruction for SBRT: (a) simple open fields (2 × 2–10 × 10 cm^2^), (b) a square wave chart pattern, and (c) the MLC position detectability test in which the MLCs were changed slightly. In clinical cases, we carried out 6 and 8 static IMRT beams for SBRT in the lung and liver. For MLC test patterns, the differences between COMPASS and MC were around 3%. The COMPASS with the dolphin system showed sufficient resolution in SBRT. For clinical cases, COMPASS can detect small changes for the dose profile and dose–volume histogram. COMPASS also showed good agreement with MC. We can confirm the feasibility of SBRT QA using the COMPASS system with Dolphin. This method was successfully operated using the new transmission detector and verified by measurements and MC.

## 1 INTRODUCTION

Recently, there has been increased clinical use of stereotactic body radiation therapy (SBRT), where extremely large doses of radiation are delivered in 1–8 fractions to one or more small targets of diseased tissue. The SBRT approach of delivering a few large doses guarantees that any error made in treatment delivery has a greater radiological impact on the patient compared to the same error made during a conventional treatment regimen. Therefore, SBRT requires robust quality assurance (QA).

The ideal verification technique for SBRT is one that is applied during patient treatment by employing either entrance or exit dose measurements. A number of publications^1^, ^2^ have suggested that online verification is a prudent step to ensure correct delivery and maintain public assurance.

For in vivo entrance dose measurements, the commercial QA platforms^3^, ^4^ which are able to correlate the delivered dose to the patient's anatomy are available. Also, the COMPASS system (IBA Dosimetry, Germany) is an in vivo dosimetry system, which provides dose–volume histograms (DVHs) based analysis for each structure.^5^, ^6^ For measurements, the system uses a new transmission detector (Dolphin, IBA Dosimetry) for in beam measurements.

Since the Dolphin detector is a new device, a detailed analysis of its accuracy is mandated. Thoelking et al.^7^ have already reported on the characterization of the Dolphin and its influence on 6 MV photon beam characteristics. They showed the increase rate of surface dose, the change in percentage‐depth‐dose (PDDs), the transmission factor, and the comparison to the clinical IMRT plan. However, those results are not specialized for SBRT, moreover, a major concern with these devices is that their large pixel size will blur the measurement of the incident SBRT fluence. It is well known that the spatial resolution of a multielement detector is limited by the size and spacing of individual detector elements,^8^ as well a large detector element will result in poor resolution, and large spacing may result in missed information. In particular, SBRT QA requires a high‐resolution detector for small fields.

In this paper, we evaluate the method for in vivo 3D dose reconstruction with SBRT using the new transmission detector developed for in vivo dose verification in intensity‐modulated radiotherapy (IMRT). Specifically, we validate the detection capability of the COMPASS transmission detector for multi leaf collimator (MLC) test patterns and clinical cases using Monte Carlo (MC) calculations.

## 2 MATERIALS AND METHODS

All measurements were performed using a Synergy (Elekta, Stockholm, Sweden) linear accelerator equipped with an agility MLC. On this linear accelerator, all measurements were carried out with 6 MV photon energy. The treatment planning system (TPS) dose calculations were performed by a Pinnacle^3^ ver. 9.2 hr (Philips Radiation Oncology Systems, WI, USA) equipped with a superposition calculation algorithm. The calculation grid size was 1.0 × 1.0 × 1.0 mm^3^ for the MLC test cases and 2.0 × 2.0 × 2.0 mm^3^ for clinical plans, respectively. Regarding the correction of the transmission detector, we registered the value of 0.91 with TPS as the tray factor.^5^

### 2.A The COMPASS system

The COMPASS system provides 1‐model‐base dose computation, 2‐measurement‐based dose reconstruction using a 2D‐array for pretreatment QA (Matrexx, IBA Dosimetry), and 3‐measurement‐based dose reconstruction using a new transmission detector for in vivo dosimetry (Dolphin, IBA Dosimetry). In this study, we evaluated the measurements‐based dose reconstruction using the Dolphin detector.

**Figure 1:**
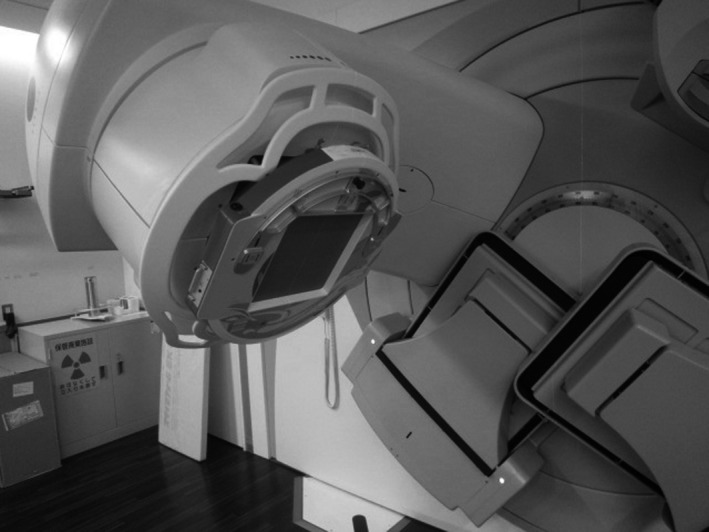
The Dolphin detector mounted on the gantry of a linear accelerator.

The COMPASS system in this study consists of the Dolphin detector and an integrated software solution comprising of a superposition algorithm^9^ that models the linear accelerator head. The 3D dose distributions were calculated from 2D measured fluence and the scatter kernel (collapsed corn) using the superposition formula. Regarding beam modeling and the reconstruction algorithm including the correction kernel, in particular, we did not modify them for SBRT because we used it for conventional IMRT and VAMT. We confirmed small fields (1 × 1, 2 × 2, 3 × 3, and 5 × 5 cm^2^) using the dose profiles for SBRT commissioning. The detector assembly is mounted in a holder attached to the treatment head of the Synergy linear accelerator (see Fig. 1). The Dolphin detector in the system provides fluence data from the measurements of SBRT treatment plans by being placed between the patient and the linear accelerator head. The fluence distribution is measured online during patient irradiation and together with the COMPASS software a detailed dose reconstruction based on the patient's CT‐dataset can be performed. The new Dolphin detector for online measurements consists of pixel‐segmented ionization chambers. This device is a 2D array of 1513 air‐vented plane parallel chambers with an active area of 240 × 240 mm^2^. The diameter of each chamber is 3.2 mm and the height is 2 mm. Up to a field size of 140 × 140 mm^2^ the pixel pitch is 5 mm which projects to approx. 8 mm in isocenter distance when measuring with a source‐to‐detector distance of 60 cm. Outside the 140 × 140 mm^2^ area the center‐to‐center distance of the chambers ranges from 5 to 10 mm. The new Dolphin detector has a higher detector density than the previous model. The beam attenuation and the increasing rate of surface dose for the Dolphin detector have already reported that they are about 10% and less than 0.1% at source‐to‐surface distance 100 cm, respectively.^7^

### 2.B MLC test cases

**Figure 2:**
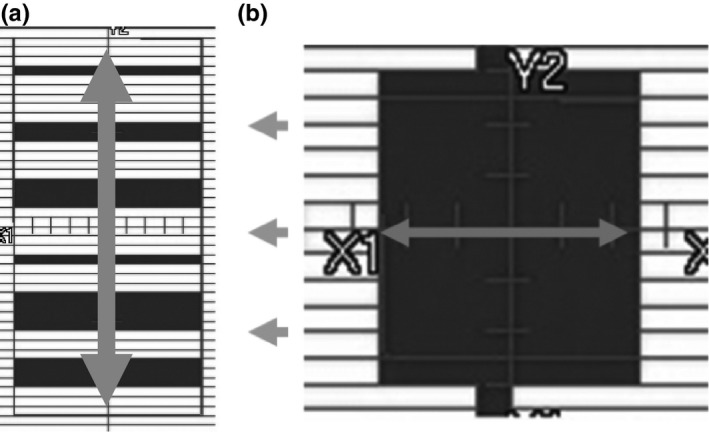
MLC test fields showing leaf positions. (a) a square wave chart pattern, (b) the fields with slightly changed MLC position. In the 5 × 5 cm^2^ open field, a systematic leaf position error ranging from 0 to 1.0 mm for one side (X1) of the MLCs was generated. All the dose profiles were acquired along the white arrows at a depth of 10 cm in a solid water phantom.

MLC test fields were set up to investigate various aspects of dose reconstruction for SBRT: (a) simple open fields (2 × 2‐10 × 10 cm^2^) for uniformity and for dose fall‐off in the penumbra region, (b) a square wave chart pattern for complex small fields, and (c) fields with slightly changed MLC positions for detectable capability (Fig. 2). In the 5 × 5 cm^2^ open field, a systematic leaf position error ranging from 0 to 1.0 mm for one side (X1) of the MLCs was generated by adjusting the major leaf offset defined inside the linear accelerator controller. All of the dose profiles were compared at a depth of 10 cm in a solid water phantom at a source–detector distance of 100 cm, along the arrows in Fig. 2. The Kodak EDR2 films were scanned 2 hr postirradiation with a flatbed scanner (ES‐10000G, Seiko Epson Corp., Nagano, Japan) with 16‐bit per depth and 150‐dpi resolution. The films were analyzed with a DD system (R‐tech, Tokyo, Japan). In the center of a 300 × 300 × 100 mm^3^ solid water phantom (Gammex‐RMI), point dose measurements were performed with a thimble type ionization chamber (PTV30013, 0.6 cc) to be able to convert the dose distribution measured with film to the absolute dose. We made the optical density‐absolute dose curve using 12 step irradiations (0–300 cGy) for the ionization chamber and EDR2 film for dose calibration.

### 2.C Clinical SBRT cases

**Figure 3:**
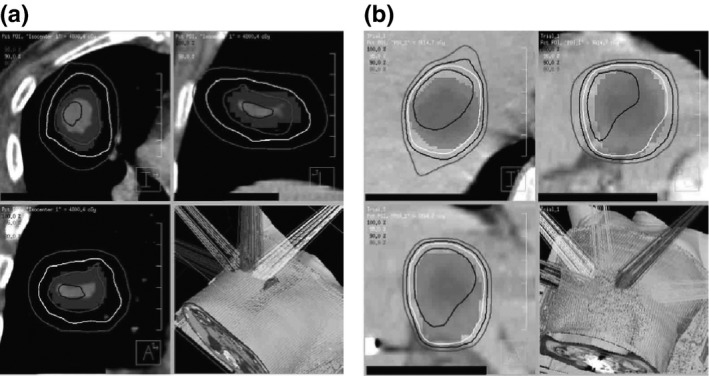
The clinical SBRT cases. (a) lung, (b) liver. The isodose lines are 80%, 90%, 95%, and 100% from the outside. A colorwash shows planning target volumes (PTV).

We confirmed the accuracy of COMPASS with Dolphin using clinical SBRT cases. We used a step and shot delivery IMRT beams for SBRT in the lung and liver (Fig. 3 and Table. 1). The prescribed doses for lung and liver are 48 Gy/4 fractions and 36 Gy/5 fractions for clinical target volume (CTV), respectively. For organs at risk (OARs), we used our hospital constraints. In clinical cases, we compared not only the dose profiles and DVHs, but also the global gamma evaluation (criteria, DTA/DD:2 mm/2%, threshold 10%).

**Table 1:** The details of the treatment plans

	Lung SBRT	Liver SBRT
Gantry (G) and couch (C) angle	6 beams (G135,C0;G180,C0;G240,C0;G300, C0; G30, C330;G345,C70)	8 beams (G30,C0;G60,C0;G180,C0;G230,C0;G290,C0;G345,C0; G20, C270;G340,C270)
Dose rate (cGy/min)	600	600
Collimator angle	0°	0°
Average filed size (cm)	4.7	5.2
Total segment	30	40
Total monitor unit	1560	864

### 2.D MC simulation

To verify the accuracy of the COMPASS system, the dose profiles and the dose distribution for lung and liver plans were also calculated by the EGSnrc/BEAMnrc^10^, ^11^ and DOSXYZnrc^12^ user‐codes. Incident photon particles were derived from the treatment‐head simulations based on the 6 MV photon beam in Synergy. In MC calculations for lung and liver plans, a voxel‐based phantom was used. The voxel‐based phantom was created by conversion of CT images into materials (air, lung, soft tissue, and bone) and mass densities. The MC dose distributions were also calibrated with the absorbed dose‐to‐water per monitor unit and multiplied by the same monitor units to the TPS for each treatment field. The calculation grid size was 2.5 × 2.5 × 2.5 mm^3^ for the dose profile and 3.0 × 3.0 × 2.5 mm^3^ for lung and liver plans, respectively. The energy threshold and cutoff were AE = ECUT = 0.7 MeV and AP = PCUT = 0.01 MeV. We confirmed that the MC was in agreement with measurements using the ionization chambers within 2% at PDDs and OCRs.

## 3 RESULTS

### 3.A MLC test cases

**Figure 4:**
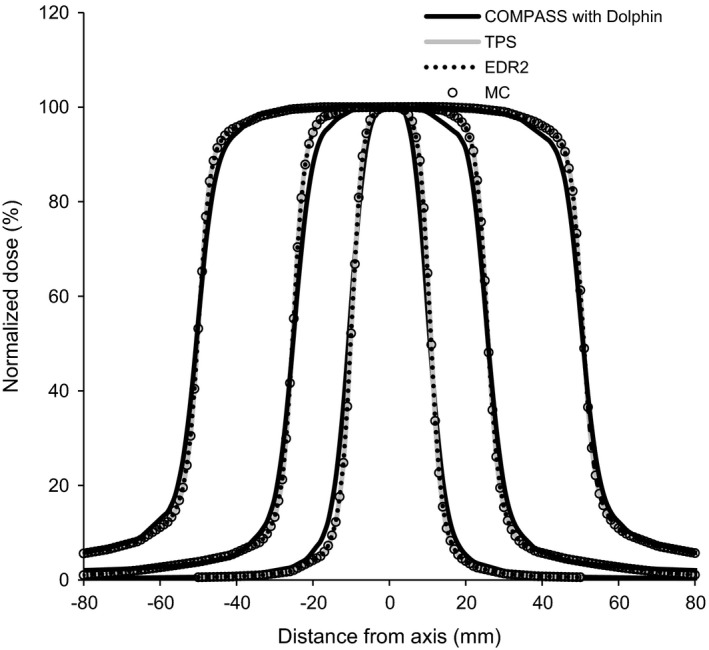
The comparison of the cross‐plane dose profiles in solid water from COMPASS, EDR2, TPS, and Monte Carlo calculations (MC) at a depth of 10 cm for 2 × 2, 5 × 5, and 10 × 10 cm^2^ square fields.

Figure 4 shows the normalized central axis cross‐plane (*x*‐axis) profiles in solid water, COMPASS, TPS, EDR2, and MC at depths of 10 cm for field sizes of 2 × 2, 5 × 5, and 10 × 10 cm^2^. For all the field sizes, the MC profiles agreed within 4% of the measured profiles for all cross‐plane profiles and the in‐field data (in‐plane not shown). For simple open fields, we confirmed good agreements with the small fields in SBRT.

**Figure 5:**
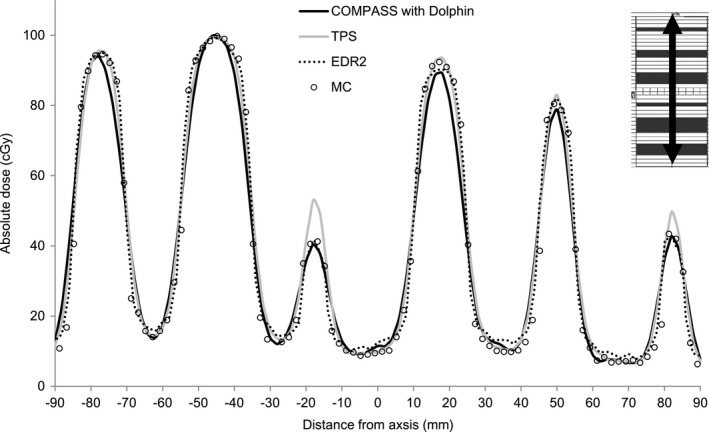
The comparison of dose profiles in solid water from COMPASS, EDR2, TPS, and MC at a depth of 10 cm for MLC test pattern.

Figure 5 shows the dose profiles in solid water from COMPASS, TPS, EDR2, and MC at a depth of 10 cm for the MLC test pattern. COMPASS, EDR2, and MC show good agreement with each other within 3% difference. TPS shows 10% difference from MC and measurements at the narrow fields. The COMPASS resulted in differences of up to 2% from MC in the MLC test pattern.

**Figure 6:**
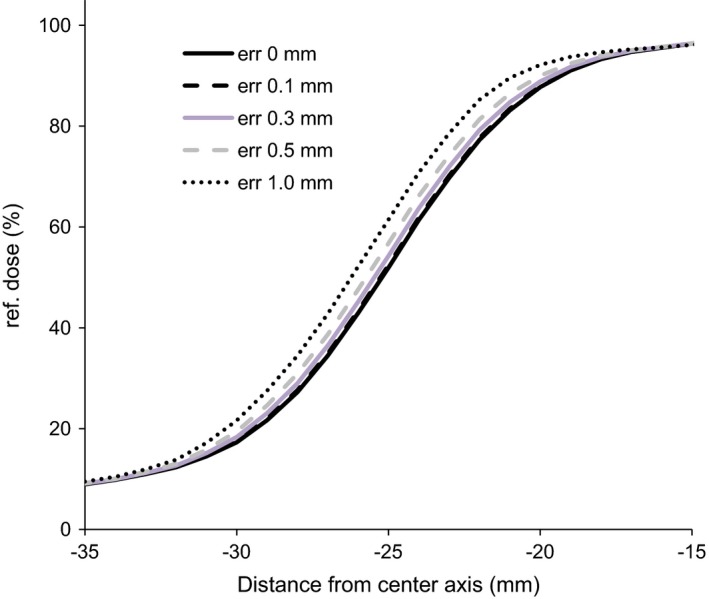
The dose profiles from COMPASS as a function of MLC positioning errors for 5 × 5 cm^2^ fields.

Figure 6 shows the detectability of MLC positioning errors. COMPASS is able to detect a difference of 0.1 mm in the MLC position. Table 2 presents the difference between COMPASS measurement and MLC positioning errors. The maximum difference was 0.24 mm at 0.5 mm of error position. Within the range of 0.1–1.0 mm, we produced MLC position errors. Those result shows that COMPASS can detect an MLC position of around 0.1 mm. The measurement uncertainties of COMPASS for MLC position errors were less than 0.24 mm.

**Table 2:** The comparison of COMPASS measurements and MLC errors

Error (mm)	0.1	0.3	0.5	1.0
COMPASS (mm)	0.27	0.47	0.74	1.23
Difference (mm)	0.17	0.17	0.24	0.23

### 3.B Clinical SBRT cases

**Figure 7:**
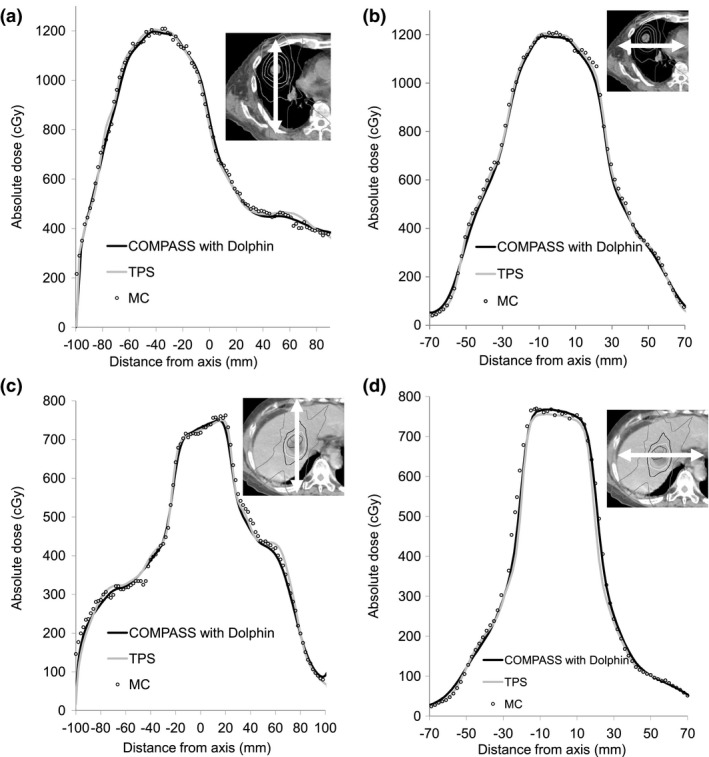
Comparison of SBRT dose profiles between COMPASS, TPS and MC calculations at an isocenter plane. The top line (a), (b) shows dose profiles for the lung case. The bottom line (c), (d) shows dose profiles for the liver case. The left side (a), (c) shows dose profiles for the vertical direction on the axial image at the isocenter. The right side (b), (d) shows dose profiles for the lateral direction on the axial image at the isocenter.

**Figure 8:**
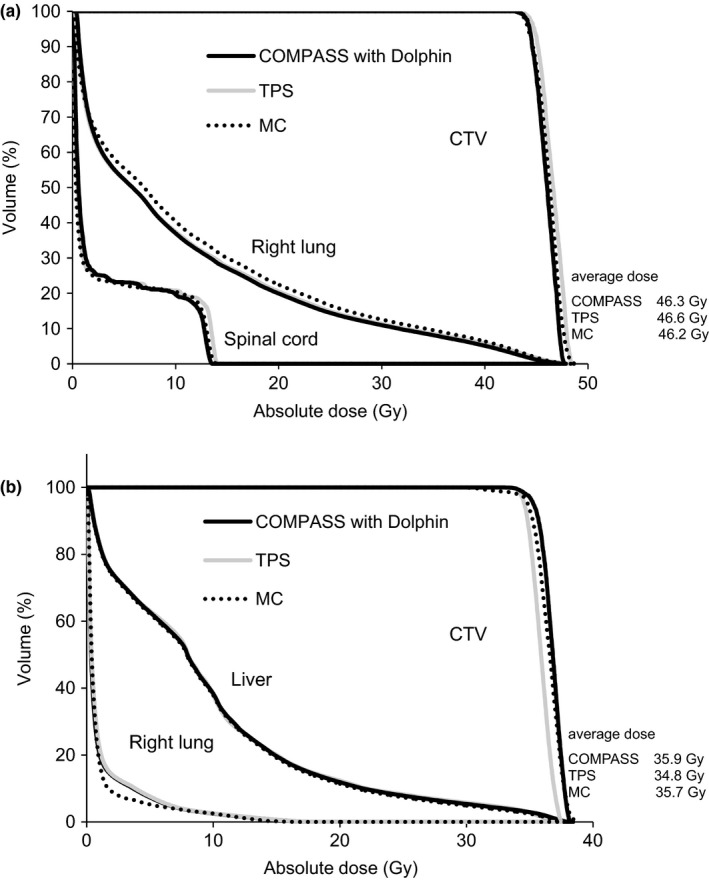
T Comparison of DVHs between COMPASS, TPS, and MC for (a) lung SBRT and (b) liver SBRT case.

Figure 7 presents dose profiles at an isocenter plane for lung and liver cases. Figure 8 also presents a comparison of DVHs between COMPASS, TPS, and MC for lung and liver cases. For the dose profiles and DVHs, there were no clear differences between COMPASS, TPS, and MC. In dose profiles, the COMPASS and TPS were in agreement with MC within 4%, except in the field penumbras. However, TPS showed a slight narrow field [Fig. 7(d)]. TPS in DVH for liver SBRT also showed an underestimation of the clinical target volume (CTV). In both cases, COMPASS showed good agreement with MC in CTV. Regarding gamma evaluation, we compared the COMPASS and MC (reference, TPS) using global gamma evaluation (criteria, DTA/DD:2 mm/2%, threshold 10%) for lung and liver cases. For the lung case, the 2D pass rates for the COMAPSS and MC were 97% and 98% at the coronal planes of isocenters, respectively. As well as in the liver case, the 2D pass rates for the COMAPSS and MC were 99% and 98% at the coronal planes of isocenters, respectively. As well as the gamma pass rate, we confirmed other gamma parameters (maximum and deviation gamma value, 3D gamma, etc), however, there was no significant difference between the COMPASS and MC.

## 4 DISCUSSION

Modern radiotherapy treatment techniques, such as SBRT, require accurate and efficient QA due to their increased complexity. In this study, the accuracy of a new transmission detector for in vivo verification in SBRT was evaluated.

### 4.A MLC test cases

Thoelking et al.^7^ reported that the influence of the Dolphin detector in treatment beams is negligible except as an output factor. They also reported the accuracy of dose distributions from the COMPASS system for MLC test patterns, and clinical cases. Because the evaluation method is the pass rate of the gamma evaluation, the detailed accuracy is unknown, but it shows highly accurate results. Our results for open fields, MLC test patterns and clinical cases also showed good agreement with MC. For the COMPASS system with the Dolphin detector, we believe that clinical use will be uneventful.

Regarding resolution, Korevaar et al.^13^ provided the same complicated MLC test pattern using the COMPASS system with a MatriXX detector (IBA Dosimetry). They reported that the differences between COMPASS and EDR2 measurements were up to 2%. This is similar to our results. Thoelking et al.^14^ also reported error detection capability about the COMPASS with Dolphin system using the gamma method for clinical cases. They concluded that the system could detect geometric errors of 1 mm. However, the results may change due to the difficulty of the treatment plan and the tolerance of the gamma method. They evaluated the general detectability in the system. Because we evaluated it using dose profiles with simple fields, we were able to evaluate the potential detectability of a Dolphin system. Many publications^15^, ^16^ have reported the accuracy and resolution of COMPASS with the MatriXX detector. The COMPASS system has detectability smaller than the physical size of the detectors using the reconstruction method. As well, COMAPSS with Dolphin and MatriXX have almost the same detectability and accuracy.

A disadvantage of 2D detector arrays is that their resolution is generally much lower than what is obtainable with film and MC. The work of Opp et al.^17^ suggests that a 5 mm resolution (at an isocenter) is sufficient to detect errors in IMRT delivery. Also, Asuni et al.^18^ reported the spatial resolution of the COMPASS transmission detector using narrow slit fields. Although the detector was the previous version (Ver. 2) where the distance between the detectors is larger than the Dolphin, the COMPASS system showed acceptable resolution for IMRT. However, for SBRT, higher resolutions are required. The detection of slight MLC position errors is essential in SBRT due to the use of smaller fields.

For MLC error position tests, the absolute values of errors were 0.24 mm which includes systematic errors. The accuracy of the static MLC position for the Synergy linac is around 0.1 mm.^19^ Therefore, considering the systematic error of MLC, the detectability of a COMPASS system is around 0.1–0.2 mm. Our results suggest that COMPASS with Dolphin has sufficient resolution for SBRT QA.

### 4.B Clinical SBRT cases

Any QA tool for SBRT requires low dependencies such as high dose rates and high doses. The Dolphin detector consists of ionization chambers. An advantage of ionization chambers is that they have less dependency. However, an ionization chamber array raises concerns about the resolution due to a volume effect.^20^ COMPASS showed good agreement with MC for clinical SBRT cases, indicating sufficient resolution. COMPASS for clinical cases can detect small changes for dose profiles and DVH. This is because the COMPASS system employs a reconstruction method combining sensitive ionization chambers and accurate beam modeling.^15^

Regarding the gamma evaluation, we think that there is no clear difference between COMPASS and MC due to small radiation fields and small targets.

For in vivo dosimetry, there are some ideas such as EPID, log‐file, and fluence measurement based on dose reconstruction. The EPID and the log‐file system are easy to use, and effective. However, the EPID shows some dependences. Also, the log‐file system is not dosimetry and log‐files include an electrical delay. A QA procedure for COMPASS is a little troublesome compared to the other two methods. However, for accuracy and robustness, the fluence measurement‐based system using an ionization chamber is advantageous.

Recent radiotherapy, SBRT is performed on many sites due to the outcomes.^21^, ^22^ But, the number of fractions is smaller, and the delivered dose is larger than in conventional treatment. Therefore, QA during treatment is very important. Sharma et al.^23^ reported that dynamic MLC (DMLC) output factor, which is a MLC positioning test, was reproducible within ± 0.5% over a period of 14 months. Monitoring is the best QA method for SBRT because the position of the MLC changes during treatment. We can confirm the feasibility of SBRT QA using the COMPASS system with Dolphin. We suggested a method for in vivo 3D dose reconstruction for SBRT. This method was successfully implemented using a new transmission detector and verified by measurement and MC.

## 5 CONCLUSIONS

We have implemented a method for in vivo 3D dose reconstruction for SBRT using a new transmission detector. In a phantom study, the differences between COMPASS and MC were around 2–3%. The COMPASS system showed sufficient resolution in SBRT. For clinical cases, COMPASS can detect small changes for dose profiles and DVH. The COMPASS system also showed good agreement with MC. Finally, we confirmed the feasibility of using the new COMPASS system with the new transmission detector for SBRT.

## ACKNOWLEDGMENTS

The authors thank Mr. Lin Xu (IBA Dosimetry, Beijing, China), Mr. Steven Ko (IBA Dosimetry, Beijing, China), Ms. Kuan‐Chuan Yeh (IBA Dosimetry, Beijing, China), Mr. Shunji Saiga (Toyo‐medic, Fukuoka, Japan), and Mr. Yasuo Takanashi (Toyo‐medic, Tokyo, Japan) for their help and discussion during this work.

## CONFLICT OF INTEREST

The authors declare no conflicts of interest.
